# Tracking the Quality of Care for Sick Children Using Lot Quality Assurance Sampling: Targeting Improvements of Health Services in Jigawa, Nigeria

**DOI:** 10.1371/journal.pone.0044319

**Published:** 2012-09-27

**Authors:** Edward Adekola Oladele, Louise Ormond, Olusegun Adeyemi, David Patrick, Festus Okoh, Olusola Bukola Oresanya, Joseph J. Valadez

**Affiliations:** 1 Liverpool School of Tropical Medicine, Pembroke Place, Liverpool, United Kingdom; 2 Lautech University Teaching Hospital, Osogbo, Osun State, Nigeria; 3 National Malaria Control Program, Federal Ministry of Health, Abuja, Nigeria; Tulane University School of Public Health and Tropical Medicine, United States of America

## Abstract

**Background:**

In Nigeria, 30% of child deaths are due to malaria. The National Malaria Control Program of Nigeria (NMCP) during 2009 initiated a program to improve the quality of paediatric malaria services delivered in health facilities (HF). This study reports a rapid approach used to assess the existing quality of services in Jigawa state at decentralised levels of the health system.

**Methods:**

NMCP selected Lot Quality Assurance Sampling (LQAS) to identify the variation in HF service quality among Senatorial Districts (SD). LQAS was selected because it was affordable and could be used by local health workers (HW) in a population-based survey. NMCP applied a 2-stage LQAS using a structured Rapid Health Facility Assessment (R-HFA) tool to identify high and low performing SD for specified indicators.

**Findings:**

LQAS identified variations in HF performance (n = 21) and enabled resources to be targeted to address priorities. All SD exhibited deficient essential services, supplies and equipment. Only 9.7% of HF had Artemisinin-based Combination Therapies and other first-line treatments for childhood illnesses. No SD and few HF exhibited adequate HW performance for the assessment, treatment or counselling of sick children. Using the IMCI algorithm, 17.5% of HW assessed the child’s vaccination status, 46.8% assessed nutritional status, and 65.1% assessed children for dehydration. Only 5.1% of HW treatments were appropriate for the assessment. Exit interviews revealed that 5.1% of caregivers knew their children’s illness, and only 19.9% could accurately describe how to administer the prescribed drug.

**Conclusion:**

This R-HFA, using LQAS principles, is a rapid, simple tool for assessing malaria services and can be used at scale. It identified technical deficiencies that could be corrected by improved continuing medical education, targeted supervision, and recurrent R-HFA assessments of the quality of services.

## Introduction

Malaria is the leading cause of child mortality in Sub-Saharan Africa. In Nigeria, malaria is responsible for 25% of infant and 30% of childhood mortality, and 11% of deaths among pregnant women [1]. To combat this heavy burden, the health system needs to ensure effective service delivery to vulnerable groups. To achieve this objective, countries need improved data systems to monitor and evaluate health system performance. This requisite is widely accepted and promoted in the international community [2].

As part of efforts to address the malaria burden, the Federal Government of Nigeria designed the Nigeria Malaria Booster Program (NMBP) and received $180 million funding during 2006 from The World Bank Group. A core objective of the program was the delivery of health interventions targeting improvements in child and maternal health for a range of illnesses including malaria [3]. Amidst several issues, the need to improve malaria case management was reinforced by increasing evidence of over-diagnosis and treatment of malaria associated with presumptive treatment [4], and due to the risk of drug resistance combined with a high cost of Artemisinin-based Combination Therapies (ACTs) [5]. Therefore, NMCP designed a system for assessment of the quality of care for malaria case management.

The NMBP monitoring and evaluation plan included a rapid health facility assessment (R-HFA) survey to obtain a baseline evaluation of the infrastructure, human resources, equipment, supplies, medications and quality of services delivered in health facilities (HF) and in Jigawa state, an add-on qualitative diagnostic assessment to give insight into findings from the R-HFA. LQAS principles were applied to the R-HFA in Nigeria as outlined in [6]. Lot Quality Assurance Sampling (LQAS) is an established [7] classification method [8] adapted from Dodge and Romig’s quality control work developed for industry in the 1920s [6], [9]. Since the time it was introduced into public health during the 1980s it has been used several times for quality assurance of health programs, including appraising health facility (HF) quality [10]. This paper presents the results of the baseline R-HFA in Jigawa state and insights from the follow-on qualitative assessment, using LQAS methodology to identify priority problems targeted for improvement. An LQAS sampling design was used in order to minimize data collection and yet classify administrative areas by the quality of HF services with a known and acceptable level of error.

## Methods

### The Instrument

We developed the R-HFA [11] indicators using the Nigerian national guidelines for implementing the IMCI algorithm; they are therefore consistent with recommendations of the World Health Organisation [3]. The R-HFA was adapted specifically to assess the quality of paediatric malaria and other child health services at HF in Jigawa. It is a four-module survey assessing health worker (HW) clinical skills for treating sick children, available infrastructure and material (e.g., medications and equipment), management and training systems, and the essential knowledge of clients when exiting HFs.

### What LQAS Is

Lot Quality Assurance Sampling (LQAS) is a classification method originally described by Dodge and Romig in the 1920s [9], which together with the work of Shewhart [12], grew to what is today called Statistical Quality Control. Their idea of sampling manufactured goods became even more critical in the subsequent war effort, where destructive testing of equipment made the concept of statistical sampling and inference essential [13]. In the industrial context an assembly line supervisor examines a small random sample from a lot of recently manufactured goods. If the number of defective goods in the sample exceeds a predetermined number, the lot is rejected as having too low a proportion of quality goods. Otherwise it is accepted. The predetermined allowable number of defective goods is called the *decision rule*. To apply LQAS the user must define a production standard, a sample size and an acceptable classification error.

During the 1980’s LQAS made the transition to international public health sciences gaining popularity as an assessment tool in a wide range of settings (see [7] for an extensive survey of applications).

### Sampling Design

The first stage of this two-stage LQAS sampling design, samples HF in each of three Senatorial Districts (SD) of Jigawa. The purpose is to classify SD as being in one of two classes labelled as “acceptable” and “unacceptable”. This classification uses the indicators in each of the four R-HFA modules (Supplementary Information S1, Figure S1). In the second stage, a sample of paediatric patients (who presented with fever, cough with difficulty in breathing, or diarrhoea–with or without blood) receiving care was used to classify the quality of services provided by an individual HW caring for paediatric malaria and other child illnesses; the knowledge of their caregivers about the child’s diagnosis, possession of a script or medication, and use of the medication was assessed when exiting the HF. This two-stage design has been used in other HFA [6], [10], [14]. After classifying HF and SD by their quality of services, we combine information across all of the SD so as to measure the prevalence of a particular indicator for the State of Jigawa as a whole. This can be done either as a stratified sample, see [15], [16] for examples, or as a cluster sample [17]. The current study uses stratified random sampling.

### Using LQAS to Classify HF in each SD

Jigawa has 606 health facilities including: 584 primary level healthcare centres (mostly health posts and dispensaries), 21 secondary level facilities and one tertiary Federal Medical Centre. The Jigawa LQAS sampling frame included 28 HF meeting the inclusion criteria of being either: primary health centres (public or private), comprehensive health centres, or secondary health facilities [3]. Eligible HF within Jigawa comprised 9HF within North East (NE) SD, 10 within Central (C) SD and 9 within North West (NW) SD (Table 1).

**Table 1:** Jigawa State with Three Senatorial Districts by Total Number of Health Facilities (HF), PMVs, Sample Sizes, and Alpha and Beta Errors for an LQAS Decision Rule of “d” = 5.

Senatorial District	Total Number of HF (N)	Sample Size of HF (n)	Alpha Error	Beta Error
Central	10	7	<0.001	0.083
North East	9	7	0.042	0.051
North West	9	7	0.042	0.051

LQAS classified SD as high or low HF performance relative to a predetermined standard by using a decision rule “d” that optimises identification of low performance SD. For each SD, a sample of “n” HF is evaluated, and a “d” was selected that determines the cut-off number of HF with adequate performance below which the SD is classified as low performance for a specified indicator. The decision rule “d” depends on the sample size, the thresholds for classifying high and low performance, and the selection of two misclassification errors: the probability of misclassifying an area with high coverage as low (α error) and the probability of misclassifying an area with very low coverage as high (β error). SD with intermediate performance are classified as high or low depending on how close they fall to the relevant thresholds. Due to the small finite universe of HF present in Jigawa, we used the hypergeometric rather than the binomial to calculate sample sizes [3]. The upper threshold “p_U_” is 80% for identifying high (or acceptably) performing SD while the lower threshold, “p_L_“ is 50%. The sample size “n” and decision rule result in α errors of ≤10%, and β errors ≤10%.

**Figure 1:**
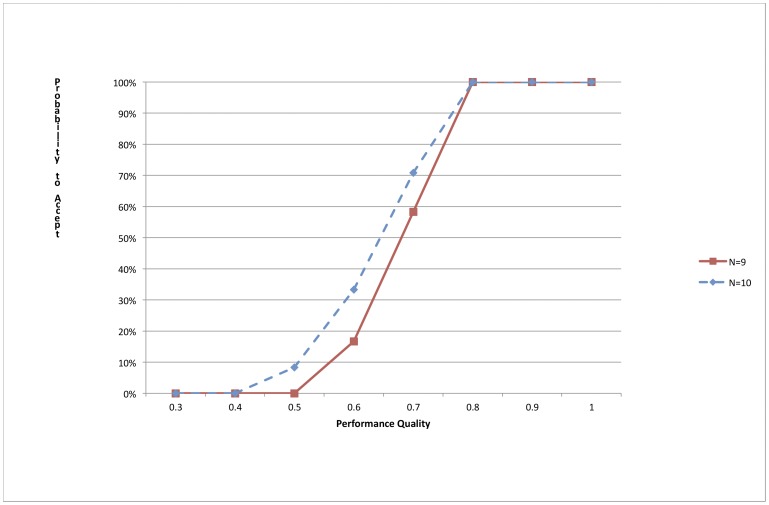
Operating Characteristic Curve for Health Facility Assessments with n = 7 and a Decision Rule of 5 for P_U_ = 80% and P_L_ = 50% for areas with a total of 9 and 10 health facilities.

These conditions yielded an optimum sample size of 7 HF in each Jigawa SD and a decision rule of 5. This decision rule has 0% alpha and 0% beta errors at the classification thresholds for NW and NE-SD, and 0% alpha and 7% beta errors for C-SD (see Figure 1 for the Operating Characteristic Curve). Simple random sampling without replacement was used to select the 7 HF in each SD. Further detail on LQAS methodology is provided elsewhere [6]. The total sample of HF in Jigawa is n = 21, which included 11 PHCs and 10 secondary facilities.

### Assessment of Individual HW Performance

In each sampled HF the most experienced HW was selected thereby resulting in an assessment showing the best case scenario. This design was selected as the results are compelling for government, since the average quality of HFs could be of lower quality. Evaluation of the selected HW’s performance in the R-HFA required a second-stage sample. In this stage the HW is the “lot” and observed delivering services to the first 6 successive patients under 5-years of age. Not more than one performance error for a given task was permitted for the 6 patients. This 6∶5 design has been used previously [6], [10], [14] and has 3% error for identifying clinicians who 95% of the time use approved clinical norms during service delivery, and 11% error for identifying clinicians using clinical norms 50% of the time [6], [10]. This design assumes performance quality among clinicians is bimodally distributed.

For both of these designs LQAS thresholds are set to identify HF that are among the *worst of the worst*. Because LQAS is best used to identify HF at the ends of the distribution of quality, managers can be assured with a small known error that the priority SD and HF with the lowest quality performance are so classified and can be targeted for improvement.

### Data Collection

Data collectors were experienced HW trained over 4-days during 16–19 March 2009. They collected data during 20–26 March 2009 under the supervision of a state manager and an author, after receiving written informed consent from all participants. The study was approved by the Liverpool School of Tropical Medicine Research Ethics Committee, and by the Ministry of Health in Nigeria. To assess reliability, an independent team visited one randomly selected HF with a completed R-HFA in each SD to verify the survey responses for items in the R-HFA that could not change in the interim. Discordance above 7% would result in the entire SD assessment being repeated. For all SD, no discordance above 7% occurred.

### Analysis

For a given performance indicator, the overall proportion of HF providing adequate services for Jigawa was estimated by weighting data by the number of HF in each SD using the Statistical Package for Social Sciences (SSPS Version 15). Confidence intervals were calculated using a finite population correction [18].

Performance problems were prioritized using a panel of 31 national, regional and local stakeholders. Representatives of key stakeholder groups included policy-makers, program managers, implementation officers, HW and health service users (mothers). In a structured meeting on 21 May 2009, stakeholders assigned priority scores (range: 1–5) to a maximum of 5 substandard tasks. Stakeholders weighted a maximum of the 10 tasks receiving the highest scores by their perceived ineffectiveness. Problems were ranked using the weighted score. The top three priority problem areas were then targeted for further qualitative research using in-depth interviews (IDI) and focus group discussions (FGD) with stakeholders.

Eight in-depth interviews using a pretested structured interview guide and three FGD were held between 22 May and 2 June 2009. Recorded interviews and FGD were transcribed and analysed with MAXQDA [19] using a thematic framework approach. The analysis followed a 5-stage iterative process using the hierarchy described in [20].

## Results

### Inadequate Inputs and Processes Supporting Health Worker Performance

The R-HFA detected in each SD severe shortages of essential supplies and first-line medications to support child health (Table 2). No SD passed as having sufficient basic supplies and medications with the exception of medicines for newborn sepsis and eye infections. Only 9.2% and 9.7% of HF had sufficient levels of essential supplies and first line medications to support child health, respectively. Inadequate infrastructure and insufficient staffing were observed in all 3 SD. Similarly, the information system was inadequately maintained in all SD.

**Table 2:** LQAS Classification of Health Facilities by Core Indicators in the Senatorial District of Jigawa and Coverage at the State Level during 2009.

Domain	Core indicators	Senatorial District LQAS Classification (DR = 5)*			State Coverage Proportion	Confidence Interval
		Central	North East	North West		
	**INPUTS**					
Staffing	Essential clinical staff present	3 (Low)	3 (Low)	0 (Low)	0.291	0.22.7–0.355
Infra-structure	Essential infrastructure available (e.g., overnight bed, power, functional latrine)	4 (Low)	0 (Low)	0 (Low)	0. 204	0.150–0.258
Supplies	Essential supplies to support child health available (e.g., cold box/refrigerator, scale)	0 (Low)	1 (Low)	1 (Low)	0.092	0.056–0.128
Drugs	First line medications for sick child management available (e.g., first lineanti-malarial, antibiotic for dysentery, ORS)	1 (Low)	1 (Low)	0 (Low)	0.097	0.058–0.136
Drugs	First line medication for newborn sepsis & eye infections (e.g., chloramphenicol)	6 (High)	5 (High)	4 (Low)	0.719	0.626–0.812
	**PROCESSES**					
Information System	Maintain up-to-date records of sick U5 children/ANC services and show evidenceof data use	1 (Low)	3 (Low)	1 (Low)	0.235	0.178–0.292
Training	HW reported receiving in-service or pre-service training in child health inpreceding 12 months	4 (Low)	5 (High)	3 (Low)	0.571	0.486–0.657
Training	HW reported receiving in-service or pre-service training in maternal orneonatal care in preceding 12 months	3 (Low)	4 (Low)	2 (Low)	0.429	0.353–0.505
Supervision	External supervision provided at least once in preceding 3 months	6 (High)	2 (Low)	6 (High)	0.673	0.584–0.763
	**HW Performance**					
Assessment	All essential assessment tasks made for sick child **	0 (Low)	0 (Low)	0 (Low)	0.000	0.000–0.000
Treatment	Treatment of sick children is appropriate to diagnosis **	1 (Low)	0 (Low)	0 (Low)	0.051	0.021–0.081
Counselling	Correct recommendations for administering all drugs prescribed**	1 (Low)	4 (Low)	0 (Low)	0.235	0.179–0.290
	**Care Taker Knowledge**					
Assessment	Caretakers able to report their child’s illness**	1 (Low)	0 (Low)	0 (Low)	0.051	0.021–0.081
Treatment	Caretaker able to accurately describe drug administration procedures totreat their child with drug prescribed by HW**	3 (Low)	1 (Low)	0 (Low)	0.199	0.144–0.254

* number of high performance HF out of a sample of 7 HF per SD and classification of SD as high or low performance based on a decision rule of 5.

** pass for at least 5 of 6 paediatric cases observed.

Processes supporting HW performance were weak across all SD. Although, 57.1% and 42.9% of HF had received training for child health and maternal/neonatal care, respectively, in the previous 12-months, no SD reached the 80% target for having staff with either in-service or pre-service training in maternal or neonatal care, and only NE-SD reached this standard for child health care. Sufficient external supervision procedures exist in both the C and NW-SD, but not in NE-SD.

**Figure 2:**
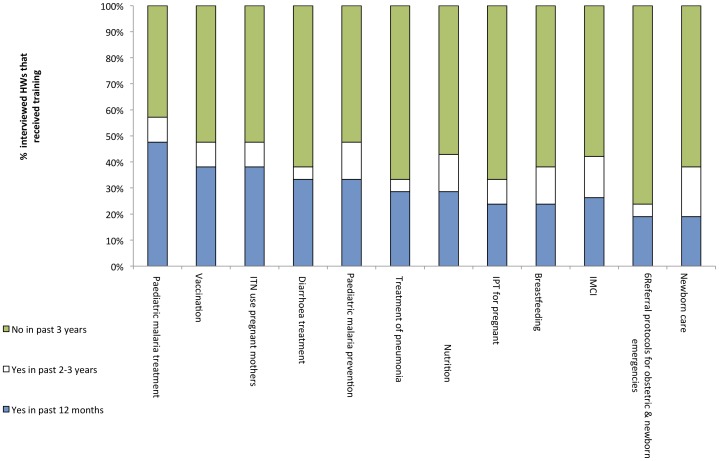
Training received by health workers in Jigawa State, Nigeria.

Subsequent IDI and FGD highlighted the poor quality and content of existing training and supervision procedures (Supplementary Information S1, Table S1). HW responses to questions about training are displayed in Figure 2. For each topic, at least 42.9% of HW had not received any form of training in the previous 3-years. The topics for which the greatest proportion of HW had received training within the last 3-years was paediatric malaria treatment (57.1%) followed by ITN use by pregnant women (47.6%), childhood vaccinations (47.6%) and paediatric malaria prevention (47.6%).

### Health Worker Performance

For all tasks concerning comprehensive treatment of the sick child, no SD passed, and only 1 HF (located in C-SD) passed. Therefore, discussion focuses primarily on the aggregate measures for the state. Overall, 126 children were seen with various illnesses of which 72.2% were diagnosed with malaria.

**Figure 3:**
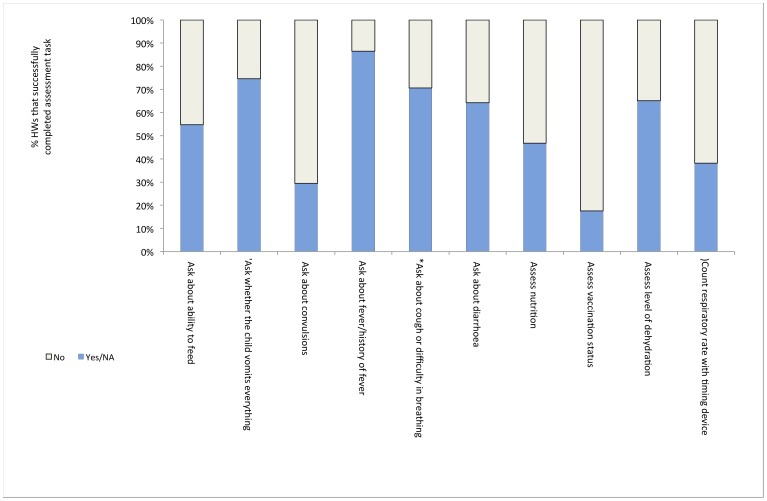
Percent of health workers in Jigawa carrying out IMCI assessment.

None of the sampled SD in Jigawa exhibited the required HW performance standard for the assessment, treatment or counselling for child illnesses as measured by the R-HFA. Figure 3 displays key HW tasks ordered by HW performance.

The assessment tasks omitted by HW included essential components of the Integrated Management of Child Illnesses (IMCI) algorithm such as assessing the child’s vaccination and nutritional status, and asking about the ability to feed (omitted by 82.5%, 53.2% and 45.2% of HW, respectively). Between 70.6% and 61.9% of HW did not complete malaria and pneumonia specific tasks such as asking about convulsions and counting the respiratory rate with a timing device. Tasks performed satisfactorily by the majority of HW include asking about fever, vomiting, breathing difficulties, and diarrhoea, and assessing dehydration.

Only 5.1% of treatments prescribed by HW were appropriate to the diagnosis. The lack of availability of first line medications is likely to be a significant contributory factor. Specifically for malaria, ACTs were prescribed in 52.7% of malaria cases diagnosed during observation of HW. A review of the last 20 medical records for under-5s in each HF showed that typically HW prescribed ACT to a smaller proportion of patients (i.e., 17.5% of malaria cases in Jigawa). While it is possible that a Hawthorne-effect is associated with the higher measure of 52.7% the fact remains that the proportion of malaria cases receiving ACT is far below the 80% threshold.

Exit interviews together with observation of HW performance revealed that counselling of mothers and caretakers by HW needs improvement. Only 5.1% of mothers/caretakers knew their child’s illness, and 19.9% could accurately describe drug administration procedures. During observation of HWs, correct recommendations for all drugs prescribed were evident in 23.5% of cases. No SD passed for these indicators. Specifically for mothers for whose children’s clinicians prescribed ACTs during observation, only 58.8% were told how to use it.

## Discussion

A population based LQAS survey had been carried out during 2006 to inform the NMCP’s project design, which included mass distribution of Long Life Insecticide Treated bednets and Artemisinin-based Combination Therapies. By the time of this HFA very few implementation activities had been carried out, and no reforms had yet to focus on the quality of service delivery. This baseline assessment identified weaknesses that were then targeted for improvements during NMBP implementation. Results highlight several weaknesses in supplies, equipment, infrastructure and human resources in Jigawa HF. NMCP identified low quality HW performance in the assessment, treatment and counselling for paediatric malaria as priorities for improvement.

### Poor access to ACTs and Other First-line Medications

ACTs and other first line medications for child illnesses were not available at the stipulated level in any Jigawa SD, despite their introduction in 2005 as the first-line treatment for malaria. Lack of access to essential medications has a significant impact on HW performance, and is likely to at least partly explain our finding of poor HW compliance in prescribing treatments appropriate to the diagnosis (5.1% of HF). This result was supported by IDI and FGD; stakeholders suggested that challenges with the supply chain management coupled with an overburdened manufacturer might account for the irregular central supply of recommended drugs to states and to health facilities. The relatively high cost of ACTs may also contribute. Client preference for drugs other than those recommended was put forward as a reason for inappropriate treatment for diagnosis. Health workers felt pressured to satisfy clients who may perceive parenteral medications as better than oral ones. Reviews of the last 20 medical records indicate that HW prescribed ACTs for treatment of 17.5% of under-5 malaria cases; this result hides significant intra-state variation (46.8%: NE-SD, 1.6%: C-SD, 9.3%: NW-SD), potentially reflecting differences in ACT availability and knowledge of clinical guidelines among the SD. Therefore, poor access to ACTs is a priority area to remedy. Strategies to address this challenge may include strengthening the commodity logistics management system in addition to making ACTs more affordable.

### HW Performance Issues

Results underscore the shortfalls in HW performance in Jigawa. Follow-up discussions with stakeholders uncovered a low level knowledge of clinical guidelines among HW, possibly because distribution of guidelines was not accompanied by training. Other studies have also found that the assessment by HW of child illnesses is often incomplete and limited to determining fever history [21], [22]. This may in part account for the high proportion (72.2%) diagnosed to have malaria during March, which should be a relatively low transmission period in Jigawa state. While inappropriate use of anti-malarials has been observed in other countries [23], [24], this did not occur in Angola where ACT stockage at HF was sufficient [21]. The need for improved counselling in Jigawa reflects findings in other settings [24]. Follow-up IDI and FGD identified lack of training and supervision, insufficient access to first-line medication and an overburdened case-load as the major causes holding back HW performance.

Critically, 42.9%, 52.4% and 66.7% of HW had not received training in paediatric malaria treatment or prevention, and IPT for pregnant women, respectively, in the previous 3-years.

Factors affecting HW performance have been extensively investigated. Studies found that training alone is ineffective for improving quality of care for malaria [25] or other illnesses including diarrhoea [26], and needs to be combined with other interventions for integrated programming. Enhanced supervision has been found to be effective, although obstacles to successful implementation exist [27]. A Kenyan study demonstrated that in-service training improves HW treatment practices if accompanied by provision of clinical guidelines and more frequent follow-up supervision [28]. Other studies also showed that training is most effective among HW who immediately use their new skills [10]. An IMCI study in Uganda showed that large-scale training is only effective if it includes clinical practice, has qualified trainers, effective follow-up supervision, policies supporting HW motivation, and essential drugs and equipment [29]. Other factors influencing HW performance include using culturally relevant materials, financial incentives [30] and an independent system of accreditation [31].

Therefore, our results indicate an urgent need for improved training and supervision for paediatric malaria, but focused on clinical practice and use of qualified trainers, and be combined with other interventions including distribution of clinical guidelines and establishing incentives as part of an integrated programmatic approach. To avoid deleterious effects of avoiding treatment of other diseases, a study in Malawi showed that training for malaria is best delivered as part of a broader approach targeting child illnesses [25], particularly in view of the disease burden associated with over-diagnosis of malaria and under-diagnosis of other febrile illnesses [5]. This finding supports Nigeria’s policy of delivering care for malaria as part of a broader package of services [3].

### Using Recurrent Rapid Health Facility Assessments

This R-HFA, using LQAS principles, proved to be a rapid, simple tool for assessing malaria services and can be used at scale. It identified practical technical deficiencies that could be corrected by improved continuing medical education, intensive supervision, and recurrent R-HFA of the quality of services in Jigawa and other Nigerian states. The advantage of inculcating LQAS into the tool was its ability to identify local variations in key performance indicators at the SD, while collecting data for State and potentially, regional and national assessments. If integrated within on-going M&E systems, R-HFA provides managers with a tool to identify gaps in human, material and infrastructural inputs in SD and to accurately target resources to areas that are most in need. The application of LQAS for HFA for improving program management has been demonstrated in other settings [32], [33]. This study illustrates how an R-HFA that uses a 6∶5 clinical observation methodology when combined with an observation checklist can be used as a practical, evidence based supervisory tool.

### Way forward

HF must remedy the lack of medicines and the lack of continuing medical education for HW. Gaps in individual HW performance could be addressed on-site by supervisors or targeted for subsequent training. Training should be part of a broad package of interventions supported by follow-up supervision [28]. These actions should lead to improvements in malaria case management.

### Limitations

One limitation of the R-HFA is that it does not assess the accuracy of diagnosis, which in this setting largely relies on clinical information. Nigeria’s switch to ACTs and the evidence of a high malaria disease burden associated with over-diagnosis of malaria has increased the importance of accurate diagnosis [5]. Potentially the LQAS 6∶5 observation rule could be applied to establish the accuracy of diagnosis within HF using RDTs. A second limitation of the R-HFA is that while it correctly identifies challenges with care in health facilities, it does not identify reasons for these challenges. Further work is needed to determine the extent of staff shortages and reasons for staff absences. This limitation was addressed in this study by follow-on qualitative assessments. A third limitation is that the methodology used here does not permit the calculation of precise prevalence indicators at the SD level as it is only intended for classification. It is only when aggregated to the State level that sufficient precision results when calculating a conventional coverage proportion.

### Conclusion

R-HFA when using LQAS is a beneficial and rapid approach for assessing the quality of health care services in large and less accessible locations such as Jigawa state. These principles can be applied to large areas with substantial savings in human resources resulting.

## Supporting Information

Figure S1 **Map of Jigawa Senatorial Districts.**
(TIF)

Supplementary Information S1 **Weighted Group Judgement and Qualitative Analyses of R-HFA Problem Areas.**
(DOCX)

Table S1 **Prioritization of Problem Areas to Address.**
(DOCX)
