# Supplementary figures and images for: Tracking the Quality of Care for Sick Children Using Lot Quality Assurance Sampling: Targeting Improvements of Health Services in Jigawa, Nigeria
Source: PLoS One. 2012 Sep 27;7(9):e44319. doi: 10.1371/journal.pone.0044319 (PMC3459971; doi:10.1371/journal.pone.0044319)

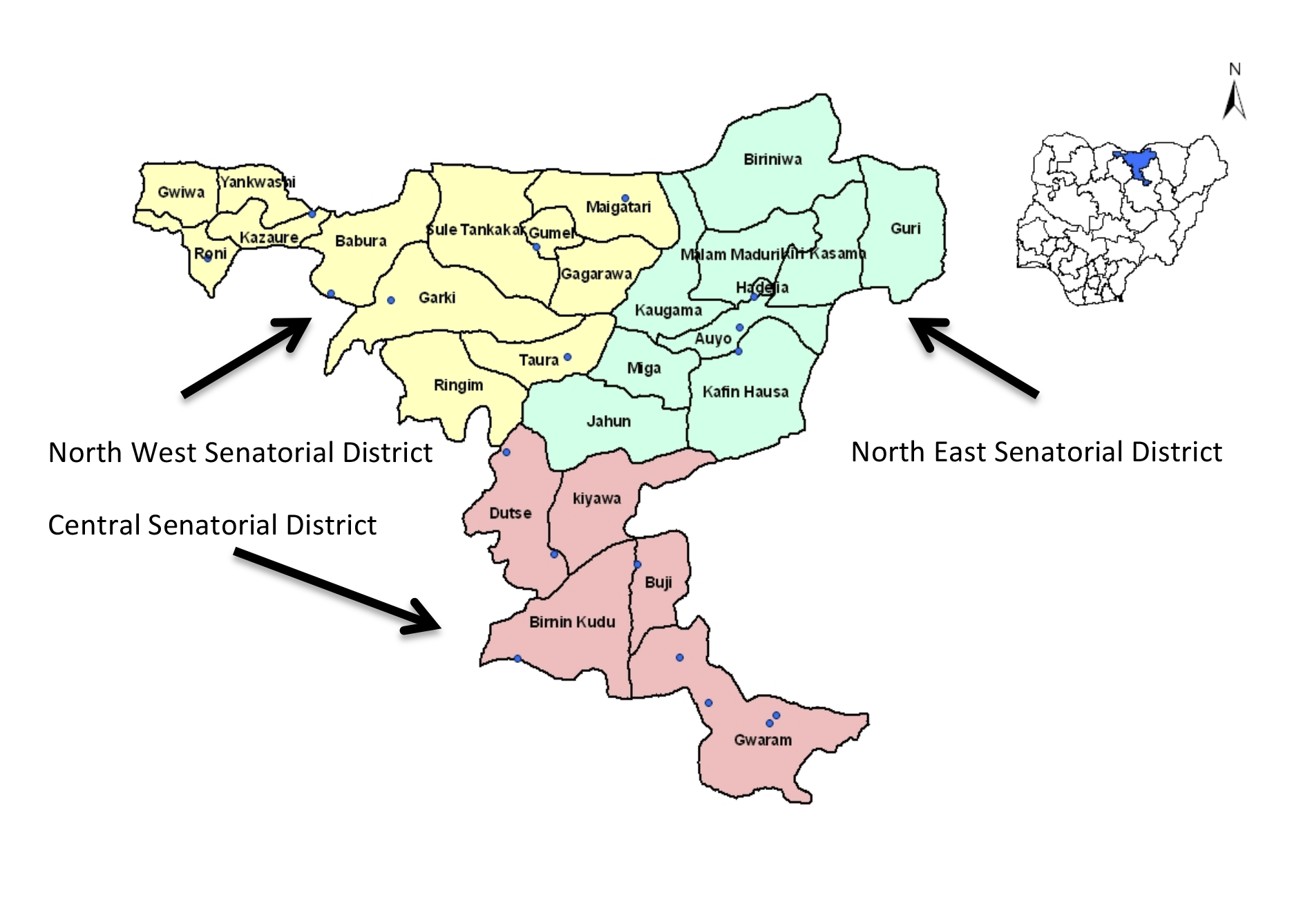

Supplement: Figure S1 — Map of Jigawa Senatorial Districts. (TIF) [file pone.0044319.s001.tif]
