# Supplementary material for: Tracking the Quality of Care for Sick Children Using Lot Quality Assurance Sampling: Targeting Improvements of Health Services in Jigawa, Nigeria
Source: PLoS One. 2012 Sep 27;7(9):e44319. doi: 10.1371/journal.pone.0044319 (PMC3459971; doi:10.1371/journal.pone.0044319)
Supplement: Supplementary Information S1 — Weighted Group Judgement and Qualitative Analyses of R-HFA Problem Areas. (DOCX) [file pone.0044319.s002.docx]

# Supplementary Information S1: Weighted Group Judgement and Qualitative Analyses of R-HFA Problem Areas

## Weighted Group Judgement

Weighted Group Judgement (WGJ) is a linear modelling technique as developed for policy making (14). Twenty-five key stakeholders were purposively selected to represent policy makers, program managers from national and state levels, implementation officers, health workers, and users of health services (mothers and other caretakers of children). In a structured setting they prioritized problems identified by the R-HFA.

Stakeholders assigned a score to each problematic intervention area with 5 being highest priority and 1 the lowest. The scores were summed to calculate a cumulative priority score for an intervention area. This score was weighted again by the coverage (as measured by the HFA) for the intervention area that created a utility score. The utility score is the WGJ used to rank the intervention areas presented in Table S1. Reasons and recommendations to improve the three top priority areas were explored through in-depth interviews and focus group discussions.

**Qualitative Analyses**

The top three priority areas identified for improvement are: incomplete child assessment and examination tasks, inappropriate treatment for diagnosis, and inadequate counselling of caregivers.

*Incomplete child assessment*

Three main reasons for *incomplete child assessment* identified from in-depth interviews (IDIs) and FGDs are: (1) Health workers did not know the guidelines because of the unavailability of guidelines at health facilities and their lack of training. The funding agency that had previously funded training had terminated its project in the state and no replacement plans existed for IMCI training. The large number of children each health worker attends to in a day had created disincentives to take health workers away from their work for training.

(2) The high volume of clients meant that health workers were under pressure to reduce consultation time by cutting short the child assessment and examination tasks. Many health workers accepted this as inevitable.

*“So in a situation where you are too much overworked, where there is too much burden on one person obviously you cannot do more than the best you can. You cannot do 100% or even 90% coverage. You can only do what you can do within your own capability within the limit of your physical and mental resource.”* **M, 50, frontline-health worker, IDI**

(3) Supervision, although frequent, was inadequate in content and quality. Aspects of performance were not picked up during supervision.

The first reason, *poor knowledge of national guidelines*, leads to prescriptions for inappropriate drugs. For example, when asked about ciprofloxacin for treating children, a manager confidently responded “*that is not recommended*”.

“...*in most cases, there is no factual, up-to-date enlightenment and information to health workers on change of ... so and so ... drugs... It is only these private pharmaceutical companies who send their representatives who come and lecture us and then go to the pharmacy department*.” **M, 50, clinician/health-facility-manager, IDI.**

*Inappropriate treatment*

A leading reason for inappropriate treatment is the *lack of recommended drugs at health facilities*. Health facilities have two lines of drug supply: free drugs available through state control programs or drugs available on a paid basis through the private sector. Free drugs are centrally procured at the national level; their supply to the state is not consistent. This deficiency was attributed to challenges with the national supply chain combined with a global supply shortage for ACTs at the time of the assessment. When the recommended free drugs were not available free through control program, health workers prescribed whatever drug was available as an alternative.

Client preference for drugs other than those recommended was also put forward as a reason for inappropriate treatment for diagnosis. Health workers felt under pressure to satisfy their clients. Some clients also complain about side effects and request alternative drugs.

*Inadequate counselling of mothers/caregivers*

Reasons identified for inadequate counselling of caregivers are: time pressure associated with high client to health worker ratio, language limitations, and inadequate training and knowledge of guidelines. As an example of poor knowledge of guidelines, there was a pervasive belief that caregivers would not understand communication, which was thought to be essential only for chronic illnesses. Also, poor staff motivation was perceived as an impediment to providing adequate counselling, due to low salary levels, insufficient supervision and incentives and discouragement associated with gaps in equipment and infrastructure.

“...*you will see there is light [electricity] there and there is no light in this hospital – that’s a problem. We use lanterns to attend to patients. What I’m saying is that it is not always that the money is the issue, the conditions of service also matters.*” **M, 50, clinician/health-facility-manager, IDI.**
