# Supplementary material for: Tracking the Quality of Care for Sick Children Using Lot Quality Assurance Sampling: Targeting Improvements of Health Services in Jigawa, Nigeria
Source: PLoS One. 2012 Sep 27;7(9):e44319. doi: 10.1371/journal.pone.0044319 (PMC3459971; doi:10.1371/journal.pone.0044319)
Supplement: Table S1 — Prioritization of Problem Areas to Address. (DOCX) [file pone.0044319.s003.docx]

Table S1: Prioritization of Problem Areas to Address

| **PROBLEM AREA** | **Priority score** | **Coverage %** | **Coverage Yet to Achieve** | **WGJ Rank (Utility)** | **Priority** |
| --- | --- | --- | --- | --- | --- |
| Low % of HF with key sick child assessment/examination tasks completed by observed HW | 102 | 0% | 100% | 102.0 | 1 |
| Low % HF in which caretakers of sick children can correctly describe how to administer all drugs prescribed | 124 | 19% | 81% | 100.4 | 2 |
| Low % of HF in which treatment of sick children by observed HW is appropriate to classification/diagnosis | 105 | 5% | 95% | 99.8 | 3 |
| Low proportion of health workers with basic pre-service training | 146 | 43% | 58% | 84.0 | 4 |
| Skewed pattern of health worker distribution | 86 | 3% | 97% | 83.2 | 5 |
| Short duration of consultations | 109 | 33% | 67% | 73.0 | 6 |
| Low % of U5 malaria cases that were reviewed in sampled health facilities and were treated with ACTs. | 109 | 42% | 58% | 63.2 | 7 |
| Low % HF in which interviewed HW reported receiving in-service or pre-service training in maternal or neonatal care in last 12 months | 81 | 43% | 57% | 46.2 | 8 |
| Low % HF in which interviewed HW reported receiving in-service or pre-service training in child health in last 12 months | 96 | 57% | 43% | 41.3 | 9 |
| Not all HF received external supervision at least once in the last 3 months | 105 | 67% | 33% | 34.7 | 10 |
